# Therapeutic implications of transcriptomics in head and neck cancer patient-derived xenografts

**DOI:** 10.1371/journal.pone.0282177

**Published:** 2023-03-01

**Authors:** Rex H. Lee, Ritu Roy, Hua Li, Aaron Hechmer, Tian Ran Zhu, Adila Izgutdina, Adam B. Olshen, Daniel E. Johnson, Jennifer R. Grandis

**Affiliations:** 1 Department of Otolaryngology, Head and Neck Surgery, University of California, San Francisco, San Francisco, California, United States of America; 2 Helen Diller Family Comprehensive Cancer Center, University of California, San Francisco, San Francisco, California, United States of America; 3 Department of Epidemiology and Biostatistics, University of California, San Francisco, San Francisco, California, United States of America; University of California, Davis, UNITED STATES

## Abstract

There are currently no clinical strategies utilizing tumor gene expression to inform therapeutic selection for patients with head and neck squamous cell carcinoma (HNSCC). One of the challenges in developing predictive biomarkers is the limited characterization of preclinical HNSCC models. Patient-derived xenografts (PDXs) are increasingly recognized as translationally relevant preclinical avatars for human tumors; however, the overall transcriptomic concordance of HNSCC PDXs with primary human HNSCC is understudied, especially in human papillomavirus-associated (HPV+) disease. Here, we characterized 64 HNSCC PDXs (16 HPV+ and 48 HPV-) at the transcriptomic level using RNA-sequencing. The range of human-specific reads per PDX varied from 64.6%-96.5%, with a comparison of the most differentially expressed genes before and after removal of mouse transcripts revealing no significant benefit to filtering out mouse mRNA reads in this cohort. We demonstrate that four previously established HNSCC molecular subtypes found in The Cancer Genome Atlas (TCGA) are also clearly recapitulated in HNSCC PDXs. Unsupervised hierarchical clustering yielded a striking natural division of HNSCC PDXs by HPV status, with *C19orf57* (*BRME1*), a gene previously correlated with positive response to cisplatin in cervical cancer, among the most significantly differentially expressed genes between HPV+ and HPV- PDXs. *In vivo* experiments demonstrated a possible relationship between increased *C19orf57* expression and superior anti-tumor responses of PDXs to cisplatin, which should be investigated further. These findings highlight the value of PDXs as models for HPV+ and HPV- HNSCC, providing a resource for future discovery of predictive biomarkers to guide treatment selection in HNSCC.

## Introduction

Head and neck squamous cell carcinoma (HNSCC) is the sixth most common cancer worldwide [1]. In the United States and Western Europe, the majority of new HNSCC cases can be attributed to oropharyngeal infection by high-risk strains of human papillomavirus (HPV+ HNSCC), while the incidence of HPV-negative (HPV-) HNSCC has decreased alongside a decline in tobacco smoking [2]. Cumulative evidence suggests that HPV+ and HPV- HNSCC are distinct entities that display some divergent genetics and clinical behaviors. In non-smokers, HPV+ HNSCC is associated with better survival and arises in younger patients compared to HPV- HNSCC [3]. Treatment-related toxicity to structures of the head and neck, especially from radiotherapy, can result in permanent life-altering disability, including impaired speech and swallowing [4]. Therefore, identifying subgroups of patients that do not require treatments associated with short-term and long-term toxicities, as well as developing effective targeted therapies with minimal side effects, is critical for maximizing function and preserving quality of life for survivors of head and neck cancer. Opportunities to reduce therapeutic toxicity are especially significant in HPV+ disease, given the increased survival of these patients and the frequent use of radiation or chemoradiation as primary therapy [1]. However, identification of clinically useful predictive biomarkers requires the availability of robust and reliable preclinical HNSCC models. The dearth of suitable preclinical HNSCC models, particularly for HPV+ HNSCC, is a major barrier to the identification and clinical translation of plausible biomarkers that identify subpopulations of patients who may require less toxic therapy to achieve durable cures.

Mice harboring patient-derived xenograft (PDX) tumors have emerged as more translationally relevant preclinical cancer models compared to mice implanted with cell line xenograft tumors, due to the closer resemblance of PDXs to the primary human tumors from which they are derived [5]. We and others previously reported that protein expression profiles of HNSCC PDXs are highly similar to the proteome of HNSCC primary tumors in The Cancer Genome Atlas (TCGA), and amplifications or mutations that define HNSCC primary specimens are typically retained in corresponding PDX models [6–8]. However, there is a paucity of reports providing detailed transcriptomic analyses of both HPV+ and HPV- HNSCC PDXs. A systematic review found that of 12 studies which compared objective characteristics of HNSCC PDXs to their corresponding patient tumors, over 80% (10/12) employed a histological analysis, compared to 50% (6/12) that compared gene expression patterns with the primary tumor, and even fewer that evaluated copy number variation (4/12) or proteomics (1/12) [9]. This gap in the characterization of HNSCC PDXs at the mRNA level is meaningful, as analysis of gene expression offers unique advantages over protein-level data. In particular, while assessment of protein expression is limited by the availability of antibodies for use in immunohistochemistry or reverse phase protein array (RPPA), RNA sequencing (RNA-Seq) offers the possibility of measuring and comparing expression of all genes across tissues of interest, as it does not rely on preselection of relevant aspects of the genome or transcriptome. For this reason, unsupervised analyses of RNA-Seq data, such as hierarchical clustering, allow for detailed subcategorization of tumor samples based on shared molecular characteristics.

To date, HPV+ HNSCC tumors have been underrepresented in studies of HNSCC PDXs, most likely because these cancers are often treated nonsurgically and biopsy material is limited for the development of such models. In addition, multiple reports suggest that the engraftment rate of HPV+ HNSCC primary tumors in immunodeficient mice is substantially lower than that of HPV- tumors [10–12], with 9 HPV+ PDXs representing the largest number reported in a single study [12]. The low number of available HPV+ PDX models, and a lack of corresponding RNA-Seq data, leaves a substantial gap in our ability to probe the transcriptomic differences that drive HPV-associated HNSCC in preclinical studies [7, 13–15]. In the present study, we performed RNA-Seq analyses on a cohort of 64 HNSCC PDXs, including 16 HPV+ PDXs, substantially expanding our current understanding of the transcriptome of HNSCC PDXs and enabling the rational selection of models for preclinical investigations.

To determine the utility of HNSCC PDX models as proxies for human disease, we compared broad patterns of gene expression between our large HNSCC PDX collection and human tumors of The Cancer Genome Atlas (TCGA), observing close concordance of well-established molecularly defined HNSCC subtypes in both cohorts. We queried the impact of mouse stromal contamination on interpretation of the PDX transcriptome, demonstrating that filtering out mouse mRNA reads had no significant impact when comparing RNA-Seq data in this PDX cohort. We conducted unsupervised clustering based on gene expression within our PDX collection, revealing a stark natural separation of PDXs by HPV status. Further transcriptomic analysis between these clusters demonstrated differential regulation of key genes between HPV+ and HPV- PDXs. Among the most highly differentially expressed transcripts by PDX HPV status was *C19orf57*, a gene previously correlated with positive response to cisplatin in cervical cancer [16]. In this PDX cohort, we demonstrate enhanced anti-tumor responses to cisplatin in HNSCC PDXs with higher *C19orf57* expression, which underscores the translational relevance of our transcriptomic findings in this PDX collection.

## Materials and methods

### PDX generation

PDXs were developed from surgical resection specimens of HNSCC tumors of 64 patients reflecting the clinical characteristics of this cancer (Table 1). All specimens used for PDX generation were obtained from patients >18 years old. Approval from the Institutional Animal Care and Use Committee (IACUC) at the University of California, San Francisco, was obtained for all animal studies (protocol #AN187611). The PDXs were derived with written consent from patients with primary HNSCC tumors under Institutional Review Board approval and were established in 5- to 6-week-old NOD.Cg-Prkdc^scid^ Il2rg^tm1Wjl^/SzJ (NSG) mice (The Jackson Laboratory, Bar Harbor, Maine), as previously described [6]. The overall engraftment rate was 79% (64/81), and the protein expression profiles of 60/64 PDXs were previously reported by our group in a 2016 publication [6]. Anesthesia was provided via inhaled isoflurane (1–5% effect), with analgesia by subcutaneous injection of meloxicam at 5 mg/kg after tumor implantation. Every effort was taken to alleviate suffering, animals were handled and treated only by trained personnel, and all animals were consistently monitored for metastasis and general decline in health.

**Table 1:** Demographic and clinical characteristics of the PDX collection.

		n (%)
Age	mean (SD)	57.9 (12.0)
Sex	Male	52 (81%)
	Female	12 (19%)
AJCC Stage	I	7 (11%)
	II	7 (11%)
	III	13 (20%)
	IV	37 (58%)
Primary Site	Oral Cavity	35 (55%)
	Pharynx	20 (31%)
	Larynx	9 (14%)
HPV status *(by sequencing)*	HPV+	16 (25%)
	HPV-	48 (75%)
HPV status *(clinical)*	HPV+	14 (22%)
	HPV-	50 (78%)

Characteristics of the 64 patients from whom the PDX collection was derived, including age, sex, AJCC stage, primary tumor site, and HPV status. HPV status was determined via two methods of classification, both sequencing-based (from RNA-Seq alignment to HPV viral genome; see methods) and a traditional clinical methodology (based on HPV ISH when available, or p16 positivity in oropharyngeal tumors). AJCC, American Joint Committee on Cancer staging; HPV, human papillomavirus; SD, standard deviation.

### RNA sequencing (RNA-Seq) and mouse read filtering

Total RNA was extracted from lysates of whole PDX tumors using the Qiagen RNeasy kit according to the manufacturer’s instructions. The total RNA was enriched for polyA transcripts and sequenced at the QB3 Genomics center at the University of California, Berkeley, using the NovaSeq 6000 platform. After initial sequencing, the unaligned reads were derived from a combination of sources including human, mouse, viral, and ostensibly other sequences. Reads were classified to a probable source prior to mapping to the human reference and expression counting. Adapters were trimmed and reads failing QC removed using fastp (v0.20.0). *Xenome* (v1.0.0), a k-mer index based pseudoaligner, classified reads as "human," "mouse," "both," "neither," or "ambiguous" [17]. Reads identified as uniquely human were advanced to the human expression pipeline. rRNA matches were removed by aligning against a ribosomal reference with bwa. The remaining reads were mapped against the Ensembl GRCh38.90 reference, and expression counts were estimated using RSEM (v1.3.1). Library sizes were adjusted using the weighted trimmed mean of M-values method from the Bioconductor edgeR package and data were transformed to log counts per million for most analyses [18]. Genes with median count less than 10 were excluded. We performed a special processing of the data to identify subtypes so that our data was on the same scale as data from The Cancer Genome Atlas (TCGA) [19]. For the subtype analysis to match the processing of TCGA data we adjusted the library size using the upper quartile method from the edgeR package. Raw and processed RNA-Seq data are openly available via the Gene Expression Omnibus (GEO) repository under accession number GSE207182.

### Determination of HPV status in PDXs

Two distinct methods to characterize HPV status were used in this study. To determine the clinical HPV status, *in situ* hybridization (ISH) for HPV on tumor tissue was utilized whenever available. For oropharyngeal tumors that did not have HPV ISH status, p16 positivity was used a surrogate marker for HPV. The p16 threshold for positivity was a 70% nuclear and cytoplasmic staining cutoff, a commonly used threshold recommended by the 2018 practice guideline from the College of American Pathologists [20]. All tumors outside the oropharynx that did not have ISH data available were assumed to be HPV- because of the limited utility of p16 as a marker for HPV-driven cancers in non-oropharyngeal tumors. The HPV status via sequencing was determined based on RNA-Seq coverage of E6/E7 within the HPV16/18 viral genome alignment for each PDX, with a count greater than 50 considered to be HPV+. This alignment cutoff was determined empirically based on the distribution of counts and to facilitate concordance with clinically-based HPV status (as described above). Only reads classified as non-human by *Xenome* were aligned in the viral pipeline, using bwa-sw against GenBank’s HPV16 (K02718.1) and HPV18 (AY262282.1) genomes. All E6/E7 expression in PDXs from this study mapped only to the HPV16 genome, with no E6 or E7 expression found in the HPV18 alignment. Two PDXs were positive for HPV by sequencing but negative clinically (PDXs 27 and 41), and HPV ISH was not available for either. One patient had a base of tongue SCC that was p16-negative, while the other had SCC of the oral tongue (which stained positive for p16 but was assumed to be “clinically negative” due to positioning outside of the oropharynx). For the ultimate designation of HPV status in our PDXs for further analysis, the sequencing classification was chosen over clinical status to best identify tumors with objective evidence of HPV-mediated carcinogenesis.

### Comparison of PDX and TCGA expression data

#### Subtype assignment

For the determination of molecularly-defined HNSCC subtypes in our PDXs, we started with the 279 patient samples (and corresponding 718 genes) from TCGA that had subtypes assigned [19]. These subtypes were basal, mesenchymal, atypical and classical. The expression data was used as received, after median-centering it. We calculated the centroids for each of the subtypes. We then matched our transcripts to TCGA data using gene name or Entrez gene ID and were able to come up with 679 total matches. For each of our samples we calculated the Pearson correlation coefficient with each of the TCGA centroids, and then assigned each sample the subtype that had the highest correlation. For visual comparison of the PDX and TCGA subtypes, the two datasets were then separately clustered using hierarchical clustering.

#### Evaluating the TCGA signature within PDX expression data

A permutation-based approach was used to assess whether the TCGA signature was represented in the RNA-Seq data from our PDXs. Subtype assignments for the PDXs were made based on correlation with the TCGA centroids, as above. We calculated the minimum Euclidean distance from each PDX sample to the centroid of samples assigned to the same TCGA subtype. Then we permuted the TCGA subtype labels maintaining the same number in each subtype and repeated the assignment and minimum Euclidean distance calculation. These distances based on permuted labels gave a reference distribution to which we could compare our actual distances. The associated p-value addressing whether the TCGA subtypes were reproduced in the PDX data was estimated as the proportion of permutation distances calculations that were greater than the distance with the real subtypes. For estimating this p-value we performed 10,000 permutations.

### Unsupervised clustering and differential expression of PDX RNA-Seq data

Hierarchical clustering of expression data from our 64 PDXs was performed based on the 2,000 most variable genes in the dataset using (1—the Pearson correlation coefficient) as the distance metric and the Ward’s agglomeration linkage method. Tests of association of sample conditions with heatmap clusters utilized Fisher’s exact test. The voom method was used to estimate differentially expressed genes between samples [21]. Adjustment for multiple comparisons utilized the Q-value method from the Bioconductor qvalue package [22]. Genes with a q-value <0.05 were considered significant.

### Assessing *in vivo* response to cisplatin

To assess for differential cisplatin response by *C19orf57* expression, two HPV+ PDXs and four HPV- PDXs were grown as described previously [6]. When the PDX tumor volume reached approximately 200 mm^3^, mice were randomized and treated with vehicle control (saline) or 5 mg/kg of cisplatin once per week by intraperitoneal injection (2–4 repeats per PDX for both vehicle and cisplatin, totaling 40 mice). Tumor dimensions were measured with calipers twice per week, with tumor volume calculated using the formula length × width × width/2. Animal health and behavior was monitored daily for wellbeing. The endpoint tumor volume for cisplatin-treated versus vehicle-treated PDXs was calculated on the final day of treatment for each PDX, which was based on tumor size in the control mice reaching a threshold which triggered sacrifice (2 cm in diameter). This endpoint was reached between day 15 and day 20 of treatment, at which point animals were promptly and humanely sacrificed via CO_2_ inhalation followed by cervical dislocation. Throughout treatment experiments, each mouse was identified using an ear tag with a unique number; the treatments and tumor measurements for each mouse were recorded according to this ear tag number. Mice in the same treatment group were housed in the same cage. No animals or data points from these experiments were excluded from further analysis.

## Results

### Filtering murine mRNA reads does not substantially impact transcriptomic analysis of HNSCC PDXs

One potential confounding element in gene expression analyses of PDX models is the presence of contaminating murine stromal cells, which surround and infiltrate engrafted human tumor samples to varying degrees. While a detailed technical workflow was published for filtering and removing mouse reads from the transcriptome of PDXs representing multiple malignancies, HNSCC was notably not included in the study [23]. To our knowledge, only two previous studies have mapped the HNSCC PDX transcriptome to the human genome while explicitly excluding mouse reads [13, 24]. One report identified a range from 65–95% of total PDX mRNA reads as human transcripts, and the other found a similar range [13, 24]. To date, the contribution of murine stroma to gene expression in HNSCC PDXs is incompletely understood.

**Fig 1:**
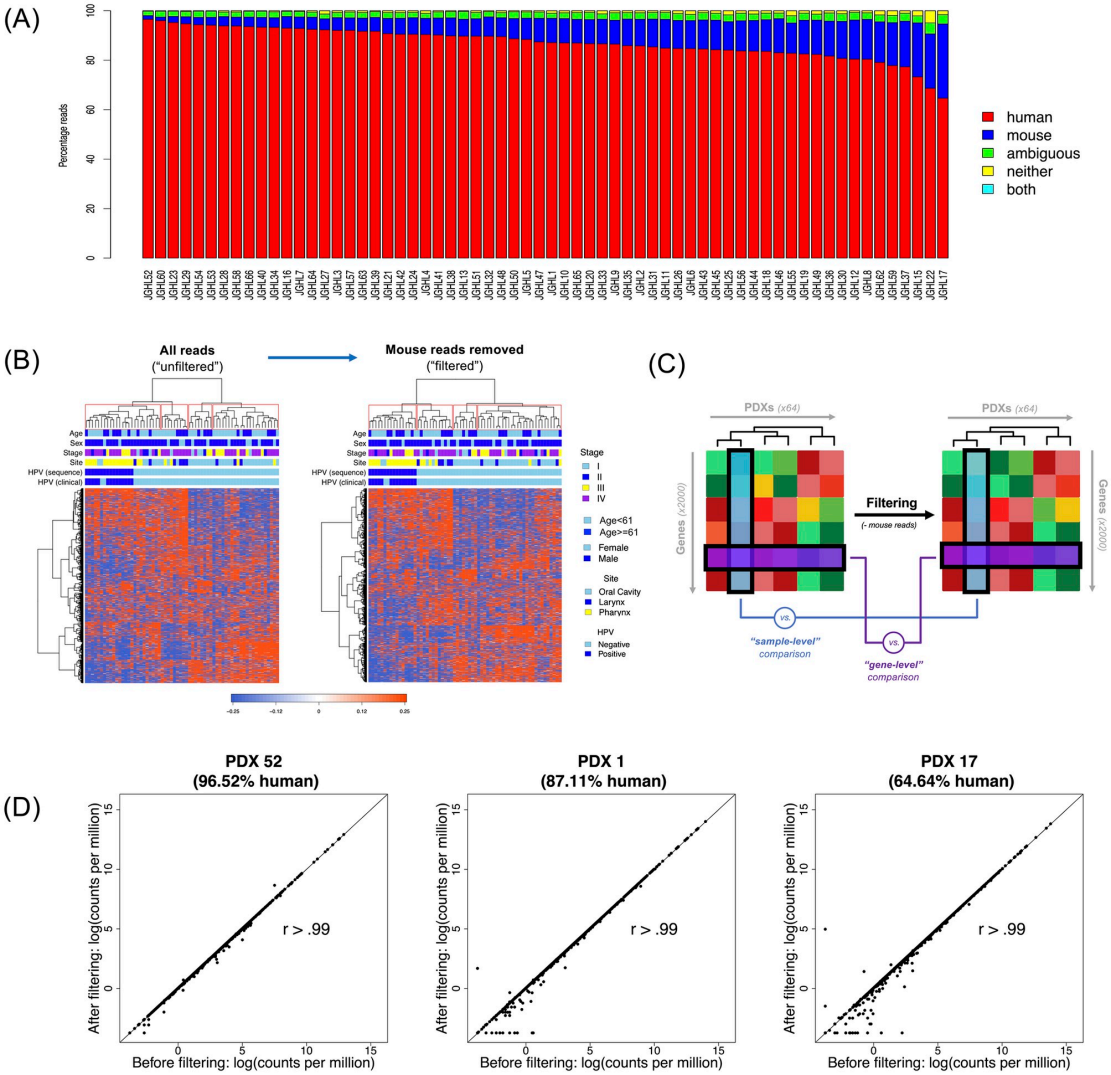
Mapping and filtering species-specific reads in the PDX transcriptome. (A) Bar Chart depicting the percentage of mRNA reads [y-axis] uniquely mapped to human (red) and mouse (blue) genomes, or ambiguous (green), neither (yellow), or both genomes (cyan) for each of the 64 PDX models in this study [x-axis]. (B) Hierarchical clustering based on the top 2,000 most variable genes between PDXs, using an “unfiltered” set with all reads (left) and “filtered” set with mouse reads removed (right). (C) Schematic of the distinction between “sample-level” (comparing clustergram columns [blue]) and “gene-level” (comparing clustergram rows [purple]) correlation comparisons. (D) Representative sample-level correlation plots from PDXs with the highest, intermediate, and lowest percentage of human reads (PDX 52, 96.52% human; PDX 1, 87.11% human; PDX 17, 64.64% human).

To determine the contribution of murine-derived stroma in the tumor microenvironment to the RNA-Seq data from our HNSCC PDXs, we employed *Xenome* to identify the proportion of PDX mRNA reads that aligned to the human or mouse genome [17]. Among the 64 HNSCC PDXs we analyzed, the average percentage of uniquely human reads was 87% [standard deviation (SD) 6.3%], with unique mouse reads representing an average of 9.5% (SD 5.5%), and the remaining reads characterized either as ambiguous or mapping to both species. The proportion of uniquely human reads ranged from 64.6% (PDX 17) to 96.5% (PDX 52) (Fig 1A).

To explore whether the presence of these “contaminant” mouse reads would meaningfully impact interpretation of human PDX RNA-Seq data, we compared the entire “unfiltered” sequencing dataset to a dataset containing only uniquely human reads (“filtered” set) (Fig 1B). We first examined the correlation between these datasets at the sample-level (i.e., unit of individual PDXs) by comparing the expression of the top 2,000 most variable genes for each PDX in the unfiltered set versus the expression of the top 2,000 most variable genes in the same PDX using the filtered set (represented by columns [blue] in Fig 1C, for a total of 64 analyses). There was extremely high correlation between the unfiltered and filtered datasets at the individual PDX sample level, with r>0.99 for all 64 PDXs. This high correlation was true irrespective of the percentage of human reads in each PDX tested, as demonstrated by highly similar correlation plots between the PDX with the highest percent of human reads (96.52% in PDX 52, r>0.99) compared to a PDX with an intermediate percent of human reads (87.11% in PDX 1, r>0.99) and that with the lowest percent of human reads (64.64% in PDX 17, r>0.99) (Fig 1D). The sample-level r values for all 64 PDXs are reported in S1 Table.

We next asked whether this exceptionally high correlation between the unfiltered and filtered datasets was also evident at the gene-level (i.e., unit of unique transcripts). To answer this question, the expression level of each of the top 2,000 most variable genes across our PDXs were individually compared before and after filtering for mouse reads (represented by rows [purple] in Fig 1C, for a total of 2,000 analyses). Overall, 99.7% of genes demonstrated r≥0.90, with 96.2% of genes possessing r≥0.99. The 3.8% of genes (76/2,000) with r<0.99 are detailed in S2 Table. When gene ontology (GO) biological process analysis was performed on these 76 genes, the only common pathway with q-value <0.05 was “skeletal system development” (p = 1.4 x 10^−5^). This biological process GO term was shared by 13/76 of such genes (S3 Table). Nonetheless, to maximize the generalizability of our data to human disease in the setting of this species-level refinement, only reads identified as uniquely human were utilized for subsequent analyses.

### PDXs recapitulate molecularly defined subtypes of HNSCC in the TCGA

**Fig 2:**
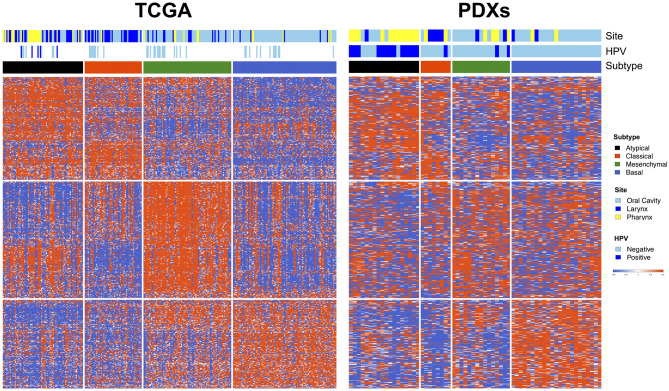
PDXs recapitulate molecular HNSCC subtypes identified in TCGA. Comparison of gene expression patterns between HNSCC samples in TCGA (left, vertical columns) and the HNSCC PDXs of this cohort (right, vertical columns), with rows representing TCGA classifier genes. The four well-characterized HNSCC molecular TCGA subtypes are conserved in the PDX collection (black, atypical; red, classical; green, mesenchymal; blue, basal).

Prior studies using direct human tumor samples have observed a natural division of HNSCC into four main molecular subtypes, defined by patterns of gene expression [19, 25, 26]. These four subtypes—basal, mesenchymal, atypical, and classical—have not only biological relevance, but also demonstrate clinically distinct behaviors including differences in recurrence-free survival [25, 26]. We sought to determine whether these established subtypes, characterized in human HNSCC tumors, were also represented in our HNSCC PDX collection. Of the 718 subtype classifier genes from TCGA (a set of previously identified genes whose collective expression signature can be used to classify a particular HNSCC tumor’s molecular subtype), 679 genes mapped to our PDX RNA-Seq data. Each PDX was grouped into the most representative subtype based on its expression signature of these classifier genes. We found that the four HNSCC subtypes (basal, mesenchymal, atypical, and classical) were similarly recapitulated in our PDXs when compared with human HNSCC tumor data in TCGA (p = 0.003 by the permutation-based test described in Methods) (Fig 2). This pronounced transcriptomic resemblance speaks to the remarkable degree to which PDX models mimic primary HNSCC. As is evident in TCGA, we also found a strong association between subtypes and HPV status, with the vast majority of HPV+ PDXs belonging to the atypical subtype (including the two PDXs which were HPV- by clinical determination but found to be HPV+ by sequencing).

### Hierarchical expression-based clustering of HNSCC PDXs reveals a natural division by HPV status and tumor site

**Fig 3:**
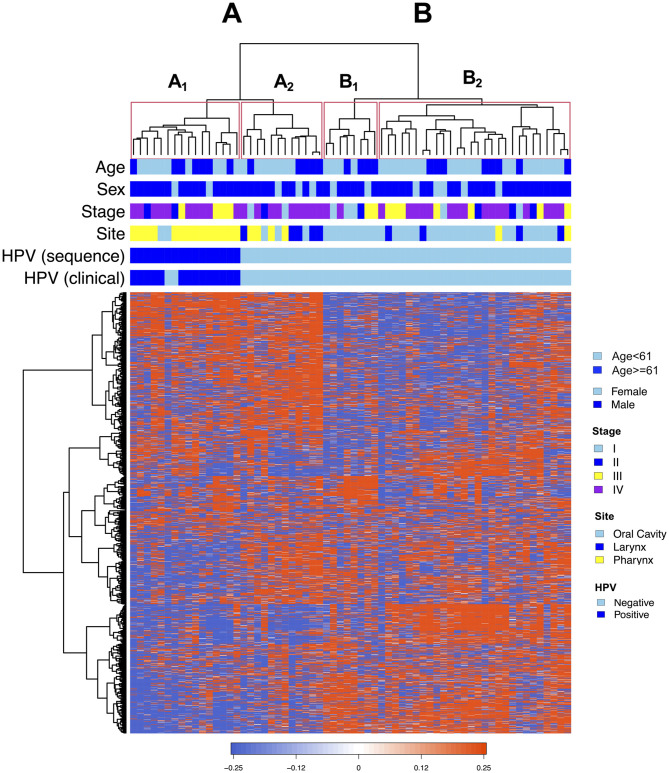
Unsupervised hierarchical clustering separates PDXs by HPV status and primary tumor site. Hierarchical clustering of RNA sequencing data via Ward’s linkage method yields two major groups of PDXs (A and B), each of which is further divided into two subgroups (A_1_, A_2_, B_1_, B_2_). PDXs (columns) align by common expression of individual genes (rows). All HPV+ tumors clustered together in a single subgroup, A_1_, which contained no HPV- PDXs. Demographic and clinical characteristics are shown above the clustergram and include age, sex, stage, site, and HPV status.

We next used hierarchical clustering of the RNA-Seq data from our PDX models to identify distinct clusters of PDXs defined by shared patterns of gene expression. Using an unsupervised approach (Ward’s linkage method) informed by the 2,000 most variable genes, the PDXs were found to organize into two main clusters (designated A and B), each of which contained two subgroups (A_1_ and A_2_, B_1_ and B_2_) (Fig 3). Cluster A was predominantly comprised of pharyngeal tumors (64.3%), compared to Cluster B which contained a majority of oral cavity tumors (83.3%). All HPV+ PDXs were located within Cluster A, where there was a further obvious division between HPV+ and HPV- tumors into two natural groupings. Notably, every HPV+ PDX in our collection clustered to a single subgroup, A_1_, which itself was comprised entirely of HPV+ PDXs. With the exception of tumor site, none of the other PDX variables assessed (including patient age, sex, or stage) were significantly associated with the expression-based clustering (S4 Table). These findings support the growing evidence that HPV+ and HPV- HNSCC display distinctive biology, and reflect the known preponderance of HPV+ tumors in the oropharynx compared with the oral cavity and larynx.

### Differential gene expression between HPV+ and HPV- PDXs has therapeutic implications

When separated by HPV status, there were 6,308 significantly upregulated genes in HPV+ compared to HPV- PDXs, and 1,862 genes that were significantly downregulated in HPV+ relative to HPV- PDXs. The 10 most significantly upregulated and 10 most significantly downregulated genes (by p-value) in the HPV+ versus HPV- PDXs are displayed in Table 2. Of these 20 transcripts, 19 genes could be identified in human HNSCC expression data from TCGA (all except *ZFPM2-AS1*). Each of the 19 genes available for comparison between HPV+ and HPV- HNSCC TCGA samples displayed concordant expression directionality as seen in HPV+ versus HPV- PDXs of this study cohort (Table 2). One of the most significantly differentially regulated genes between HPV+ versus HPV- PDX tumors was *C19orf57* (*BRME1*), which demonstrated 6-fold higher expression in HPV+ models compared to HPV- models (p = 7x10^-17^; Table 2). A previous study of TCGA patients with a variety of tumor types identified an association between high *C19orf57* expression with superior survival on cisplatin therapy compared to low *C19orf57* expression in cervical squamous cell carcinoma and endocervical adenocarcinoma [16].

**Table 2:** Top differentially expressed genes in HPV+ versus HPV-PDXs.

Overexpressed in HPV+ vs. HPV- PDXs					Underexpressed in HPV+ vs. HPV- PDXs				
	PDX cohort		HNSCC from TCGA			PDX cohort		HNSCC from TCGA	
Gene	Fold Change	P-value	Fold Change	P-value	Gene	Fold Change	P-value	Fold Change	P-value
*SMC1B*	35.5	6E-22	162	3E-108	*GPR158*	0.1	4E-16	0.1	1E-09
*SYCP2*	29.9	2E-20	45.6	4E-90	*KHDC1L*	0.1	1E-15	0.2	2E-07
*WDR76*	5.1	7E-17	2.9	9E-31	*MSX2*	0.2	3E-14	0.2	3E-19
*C19orf57*	6.0	7E-17	6.4	8E-65	*PNLIPRP3*	0.1	5E-12	0.1	1E-11
*CDKN2C*	8.2	1E-16	5.3	2E-46	*NAP1L2*	0.2	7E-12	0.3	1E-05
*ZNF541*	21.3	2E-16	54.2	5E-61	*ZFPM2-AS1*	0.3	1E-11	---	---
*RIBC2*	7.0	2E-16	8.5	2E-49	*MYO5A*	0.2	5E-11	0.3	5E-24
*TCP11*	12.6	5E-16	31.8	2E-32	*COL4A6*	0.1	1E-10	0.1	1E-12
*MEI1*	21.3	2E-15	10.6	2E-41	*PTHLH*	0.1	3E-10	0.1	3E-17
*CCNE2*	4.1	2E-15	2.9	7E-32	*CCNA1*	0.2	4E-10	0.1	2E-11

Genes significantly overexpressed in HPV+ relative to HPV- PDXs (left) and significantly underexpressed in HPV+ relative to HPV- PDXs (right). Genes in each category are sorted by most significant p-value for the comparison between fold change of HPV+ versus HPV- PDXs. Also displayed are fold changes between HPV+ and HPV- HNSCC of TCGA for these genes (with the exception of *ZFPM2-AS1*, which was not found in TCGA transcriptome).

**Fig 4:**
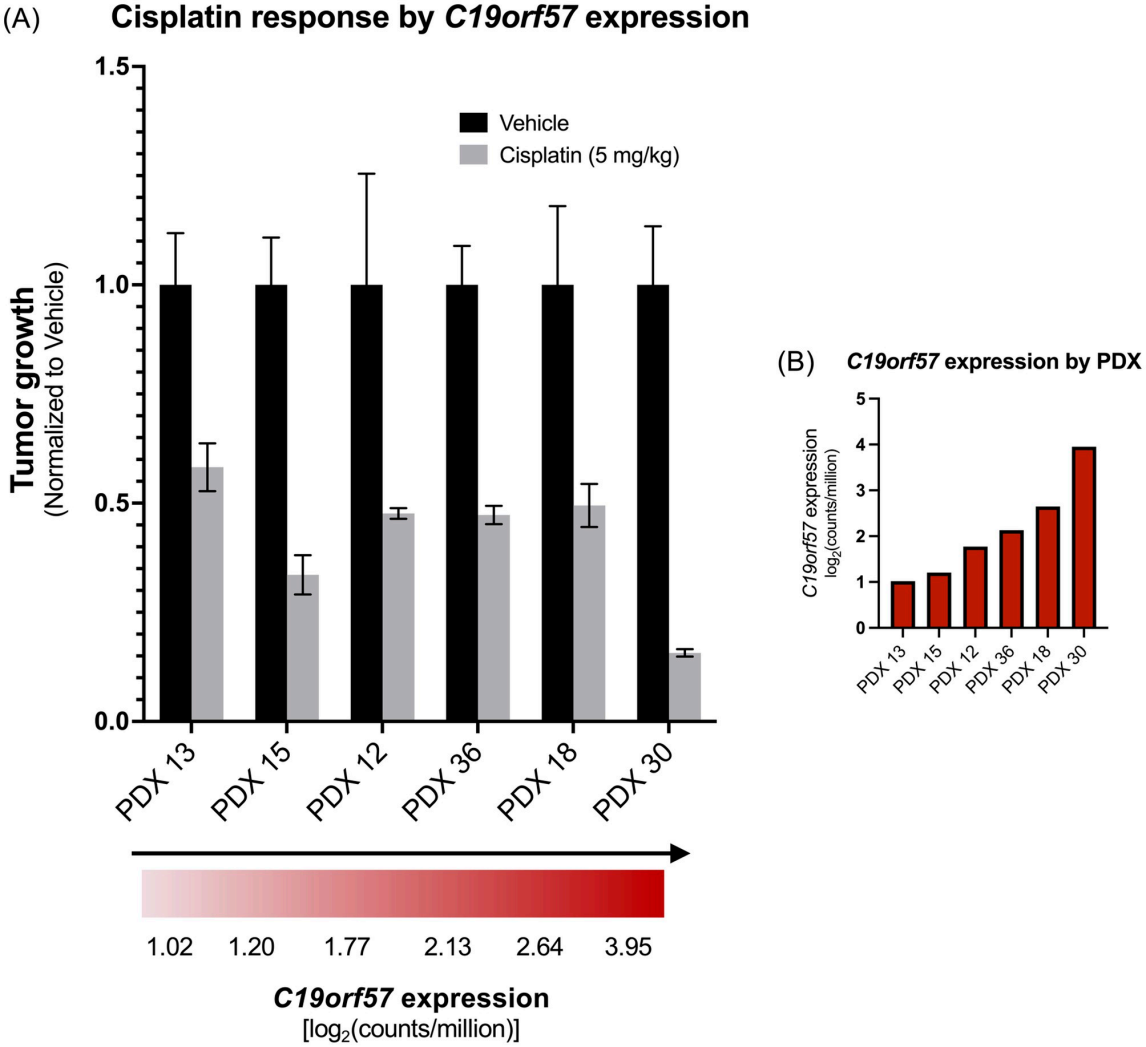
*C19orf57* expression and cisplatin response in PDXs. (A) Therapeutic response of PDXs to cisplatin, arranged in order of increasing *C19orf57* expression. (PDXs 36 and 30 are HPV+; PDXs 13, 15, 12, and 18 are HPV-). (B) *C19orf57* expression, in units of log_2_(counts/million), for each of the six PDXs tested for cisplatin response.

We next sought to investigate any potential relationship between *C19orf57* expression and *in vivo* tumor response to cisplatin in six HNSCC PDXs from our collection (four HPV- and two HPV+). The PDX that responded most robustly to cisplatin (PDX 30) also had the highest expression of *C19orf57* of those tested [log_2_(counts/million) = 3.95] (Fig 4). Moreover, PDX 30 was HPV+, and demonstrated superior cisplatin response relative to the other HPV+ PDX tested (PDX 36), which had substantially lower *C19orf57* expression [log_2_(counts/million) = 2.13]. The dramatic difference in cisplatin sensitivity at the extremum of *C19orf57* expression suggests a potential relationship which should be further investigated with larger sample sizes.

## Discussion

Developing preclinical HNSCC models that most closely reflect human HNSCC tumors is a critical step in identifying therapeutic targets and testing potential treatments for patients with head and neck cancer. While PDXs are increasingly recognized as more translationally relevant avatars compared to cell line xenografts, there are still relatively few published HNSCC PDX collections in the literature, and those that do exist have undergone varying degrees of molecular characterization [27]. In particular, the extent to which HNSCC PDXs resemble primary human HNSCC at the transcriptional level has been understudied. Here, we add substantially to the body of HNSCC PDX literature through analyses of RNA-Seq data from a large cohort of 64 PDXs (16 HPV+ and 48 HPV-). First, we interrogated the influence of contaminant mouse reads on the interpretation of PDX transcriptomic data, demonstrating that filtering mouse mRNA from RNA-Seq data of PDXs is likely an unnecessary step without tangible benefit in this PDX cohort. It is noteworthy that the lowest percentage of unambiguously human reads in a PDX within our study was 64.6%, so we are unable to conclude that mouse read filtering would be of no benefit for PDXs with human read percentages below this level. We compared the transcriptomic profile of our PDXs to gene expression profiles of human tumors, reproducing the well-defined molecular HNSCC subtypes from TCGA within our PDX collection and further supporting the utility of these models as faithful proxies for human disease. Using an unsupervised clustering approach to extrapolate patterns from the PDX transcriptomic data, we uncovered distinct PDX subtypes defined by similarity of gene expression that naturally segregated all HPV+ PDXs from HPV- PDXs. Lastly, we explored the potential clinical relevance of leveraging transcriptomic variation between our PDX models, with our data suggesting a possible relationship between *C19orf57* expression and cisplatin sensitivity, which could be investigated further.

Compared to cancer cell lines or fresh surgical specimens, the presence of murine stroma in the PDX tumor microenvironment is a theoretical limitation to the analysis of high-throughput sequencing data of both DNA- and RNA-based measurements. As a result, groups working with PDXs may opt to remove mouse-identified genetic material from that which is human-specific, using one of multiple computational methods published over the last decade (e.g., *Xenome*, *Disambiguate*, or *XenofilteR*) [17, 28, 29]. However, rigorous examination of the necessity of this step (i.e., whether species-level filtering affords tangible differences in gene expression interpretation) is lacking. Furthermore, there is often considerable variability in the proportion of species-specific reads between PDXs of the same cohort, as evidenced by a large RNA-Seq study of 95 melanoma PDXs that reported an average of 6.1% mouse-specific reads, but a range from 1% to 35% [29]. In the present study, the average proportion of uniquely human mRNA reads in our PDX tumors was 87% (range 64.6–96.5%). These results are consistent with two prior studies that filtered mouse reads from HNSCC PDXs [13, 24]. However, unlike these earlier reports, we utilized correlation analyses to determine that the process of mouse read filtering did not appreciably impact our PDX gene expression data. This lack of filtering benefit was evident both at the PDX sample-level (when comparing gene expression before and after filtering in each PDX separately) and at the gene-level (in which expression of each the top 2,000 most variable genes was individually compared across all PDXs before and after filtering). Notably, this correlation at the sample level was universally high with a correlation coefficient (r) >0.99 for all 64 PDXs tested, regardless of the percentage of human reads identified in the PDX. This suggests that a higher degree of mouse read contamination, at least in this cohort of HNSCC PDXs, does not necessarily result in greater benefit from species-level filtering. Correlation analysis at the gene level, as at the sample level, was also exceptionally high.

In this study, we demonstrate a remarkable transcriptomic similarity of HNSCC PDXs to human tumors by recapitulating four well-characterized HNSCC molecular subtypes from TCGA in our PDX models. These four HNSCC subtypes—basal, mesenchymal, atypical, and classical—were originally identified from a cDNA microarray study of 60 human tumors [25], and subsequently reproduced in two large independent cohorts of HNSCC patients [19, 26]. A similar segregation of a unified histologic cancer type into mRNA expression subgroups has also been demonstrated in squamous cell carcinoma of the lung [30]. The preservation of HNSCC molecular subtype heterogeneity in preclinical models was previously observed in a small study of 16 PDXs derived from oral cavity tumors [24], but has not been examined in PDXs of pharyngeal cancer, especially HPV+ disease. In the present work, we recapitulate the HNSCC subtypes in a substantially larger PDX cohort, including both HPV+ and HPV- PDXs. Notably, the PDXs in our study displayed a strong correlation between HPV status and molecular subtype, with the vast majority of HPV+ PDXs belonging to the atypical subtype—the same pattern seen in TCGA.

Our characterization of 16 HPV+ PDXs represents the largest reported HPV+ PDX cohort in a single study to date. Multiple groups have reported on the difficulties of successfully engrafting HPV+ tumors into mice, which is further hindered by a general scarcity of HPV+ tumor tissue to implant due to the widespread use of primary radiation or chemoradiation as curative treatment for this patient population [10, 11]. Given the overall unavailability of these models, it is paramount that HPV+ HNSCC PDXs continue to be generated and characterized, especially as the incidence of this disease entity continues to rise. In the present study, the exceptional transcriptomic resemblance of HNSCC PDXs to primary HNSCC tumors was preserved in both HPV+ and HPV- models, underscoring the utility of these PDXs for translational research.

Using hierarchical clustering analysis, we further leveraged RNA-Seq data to subcategorize our PDX cohort based on patterns of gene expression. We took an unsupervised approach without *a priori* consideration of PDX clinical characteristics to probe for overlapping transcriptomic signatures which may indicate shared biology or treatment susceptibility. This approach naturally separated our PDXs by HPV status, with every HPV+ model clustering in a single subgroup composed exclusively of HPV+ PDXs. Importantly, none of the other demographic or clinical variables assessed were significantly associated with this expression-based clustering (except tumor site, which is known to be highly correlated with HPV positivity). Such a stark transcriptomic division, identified solely based on molecular characteristics, supports the already ample evidence that HPV+ and HPV- HNSCC are distinct disease entities. This finding also reinforces the potential of harnessing PDX transcriptomics to uncover unique biologic targets in both HPV+ and HPV- HNSCC.

Analysis of differential gene expression between PDX subtypes is potentially therapeutically relevant, as RNA-Seq allows for identification of actionable alterations which may guide treatment selection. For example, one study of 37 PDXs derived from difficult-to-treat breast cancers leveraged a multi-level “-omics” approach to identify targetable vulnerabilities at the DNA (whole-genome sequencing), RNA (RNA-Seq), and protein (RPPA) levels [31]. We identified a collection of genes that were strongly differentially expressed by HPV status in the PDXs of this study, including genes with and without previously reported roles in the development of HNSCC. Of the 19 genes available for analysis in TCGA (out of 20 total transcripts identified in our PDXs), all displayed concordant expression directionality between HPV+ versus HPV- PDXs of the study cohort and HPV+ versus HPV- HNSCC of TCGA (10 overexpressed and 9 underexpressed).

Furthermore, we identified *C19orf57* as a differentially expressed gene which warrants further investigation in HNSCC patients treated with cisplatin. *C19orf57* encodes break repair meiotic recombinase recruitment factor 1 (BRME1). This protein plays a key role in meiotic recombination by facilitating homology-directed DNA repair via recruitment of recombinases such as RAD51 to DNA double-strand breaks in gametes [32]. A study that analyzed the correlation between tumor gene expression and patient survival on chemotherapy found that the top gene-drug interaction among patients with cervical squamous cell carcinoma and endocervical adenocarcinoma was that of *C19orf57* and cisplatin [16]. Specifically, high *C19orf57* expression was associated with improved survival in the setting of cisplatin therapy relative to the survival of patients whose tumors expressed lower levels of *C19orf57* [16]. This observation in cervical cancer is especially intriguing since the majority of cervical SCCs, as with oropharyngeal SCCs, can be attributed to HPV infection. The value of *C19orf57* expression in predicting cisplatin response in HNSCC, and interactions with HPV status, has not been explored.

In our study, *C19orf57* was expressed at levels 6-fold higher in HPV+ PDXs compared to HPV- PDXs, a finding consistent with two prior reports that found significantly higher *C19orf57* expression in primary patient samples of HPV+ OPSCC compared to HPV- OPSCC [33, 34]. The potential of this gene to serve as a biomarker for cisplatin response is especially compelling in the context of recent de-escalation clinical trials showing a substantial decrease in survival of patients with HPV+ OPSCC when cisplatin is omitted or substituted (such as with cetuximab) [35]. Identification of patients most likely to respond to cisplatin would minimize the toxicity and morbidity associated with platinum chemotherapy, which is vital given the younger age and improved survival of HPV+ HNSCC patients compared to those with HPV- disease. In the present study we observed that of six PDXs tested, the PDX with the highest *C19orf57* expression also demonstrated the largest response to cisplatin treatment. It is worth mentioning that this PDX had substantially higher expression of *C19orf57* than the other five PDXs tested with cisplatin, with ~2.5-fold higher *C19orf57* counts/million than the PDX with second-highest *C19orf57* expression. In addition, the PDX with the most robust cisplatin response was one of the two HPV+ PDXs tested, and responded substantially better to treatment than the other HPV+ PDX with lower *C19orf57* expression. These results suggest that HPV status may potentiate the value of *C19orf57* expression for cisplatin response prediction in HNSCC. However, there are several notable limitations to conclusions about *C19orf57* drawn from these *in vivo* PDX experiments. One limitation of our analysis is the relatively low number of PDXs tested with cisplatin in this study, and the observation that HPV+ HNSCC may demonstrate a superior baseline response to DNA-targeted therapy (e.g., radiation and platinum agents) than HPV- HNSCC, potentially due to a delay in the repair of DNA damage [36–38]. Secondly, all PDXs tested in our study responded to cisplatin (with our conclusions based on varying response degrees), so we were unable to associate low *C19orf57* expression with non-response to this agent. For these reasons, there is insufficient evidence at present to definitively implicate *C19orf57* as a predictive biomarker for cisplatin response, though our data demonstrate that analyses of the PDX transcriptome have potential for elucidating clinical response to treatment. Future studies which characterize the degree of dose-dependence between *C19orf57* expression and cisplatin response in both HPV+ and HPV- HNSCC are warranted to determine whether this transcript may hold utility in the clinical setting.

Gene expression-based stratification of HNSCC has not yet materialized as a feasible strategy for treatment selection in head and neck cancer patients. Our transcriptomic analyses of a large collection of HPV+ and HPV- HNSCC PDXs provide valuable characterization of these preclinical models at the mRNA level, demonstrating the strength of PDXs in recapitulating human biology. Our results are a valuable resource for future investigation and may be useful for identifying novel transcriptomic biomarkers of therapeutic response and new mechanistic targets in HNSCC.

## Supporting information

S1 Table Sample-level correlation between unfiltered and filtered RNA-Seq datasets. List of r values for correlation comparisons of expression of the top 2,000 most variable genes for each PDX in the unfiltered RNA-Seq dataset (mouse and human mRNA reads) versus the filtered RNA-Seq dataset (only human reads).
(PDF)

S2 Table Gene-level correlation between unfiltered and filtered RNA-Seq datasets. List of r values for the 3.8% of transcripts (76/2000) with r <0.99 at the gene-level. Correlations are comparing expression level of each of the top 2,000 most variable genes across the PDXs before and after filtering for mouse reads.
(PDF)

S3 Table Biological process pathway analysis of transcripts with lower gene-level correlation between unfiltered and filtered RNA-Seq datasets. Gene Ontology (GO) annotated pathway analysis to assess for common biological functions between the 76 genes with gene-level correlation r values <0.99. Skeletal system development was the only GO term with q-value <0.5.
(PDF)

S4 Table PDX cluster associations with patient demographic and tumor variables. Correlation of demographic and clinical characteristics of PDXs with cluster membership reveals that HPV status and tumor site are the only variables significantly correlated with hierarchical gene expression-based clustering (when partitioned by either 2, 3, or 4 clusters). Age, sex, and stage were not significantly associated with expression-based clustering in any analyses conducted.
(PDF)

## Abbreviations

*BRME1*: break repair meiotic recombinase recruitment factor 1
*C19orf57*: chromosome 19 open reading frame 57
HNSCC: head and neck squamous cell carcinoma
HPV: human papillomavirus
ISH: *in situ* hybridization
NSG: NOD.Cg-Prkdc^scid^ Il2rg^tm1Wjl^/SzJ
OPSCC: oropharyngeal squamous cell carcinoma
PDX: patient-derived xenograft
RNA-Seq: RNA sequencing
SD: standard deviation
TCGA: The Cancer Genome Atlas
