# Supplementary material for: Therapeutic implications of transcriptomics in head and neck cancer patient-derived xenografts
Source: PLoS One. 2023 Mar 1;18(3):e0282177. doi: 10.1371/journal.pone.0282177 (PMC9977000; doi:10.1371/journal.pone.0282177)
Supplement: S1 Table — List of r values for correlation comparisons of expression of the top 2,000 most variable genes for each PDX in the unfiltered RNA-Seq dataset (mouse and human mRNA reads) versus the filtered RNA-Seq dataset (only human reads). (PDF) [file pone.0282177.s001.pdf]

PDX	Sample-level correlation (r)
PDX 52	0.99975
PDX 24	0.99923
PDX 9	0.99903
PDX 55	0.99882
PDX 28	0.99841
PDX 35	0.9984
PDX 10	0.99832
PDX 50	0.99831
PDX 16	0.99823
PDX 27	0.99812
PDX 41	0.99806
PDX 42	0.998
PDX 34	0.99799
PDX 56	0.99796
PDX 59	0.99796
PDX 7	0.99795
PDX 26	0.99793
PDX 40	0.99791
PDX 25	0.99775
PDX 58	0.99774
PDX 3	0.99758
PDX 51	0.99753
PDX 65	0.99753
PDX 49	0.99749
PDX 66	0.99749
PDX 1	0.99747
PDX 57	0.99747
PDX 48	0.99746
PDX 54	0.9974
PDX 60	0.9974
PDX 39	0.99738
PDX 64	0.99734
PDX 29	0.99728
PDX 21	0.99724
PDX 45	0.99719
PDX 19	0.99711
PDX 44	0.99701
PDX 18	0.99699
PDX 23	0.99685
PDX 33	0.99682
PDX 46	0.99668
PDX 2	0.99646
PDX 63	0.99642
PDX 4	0.99641
PDX 62	0.99637
PDX 8	0.99631
PDX 38	0.99629
PDX 20	0.99624
PDX 11	0.99603
PDX 22	0.99603
PDX 43	0.99586
PDX 32	0.99583
PDX 53	0.99568
PDX 31	0.99566
PDX 6	0.99566
PDX 15	0.99554
PDX 12	0.9955
PDX 37	0.99528
PDX 5	0.99524
PDX 36	0.99512
PDX 13	0.99487
PDX 30	0.99484
PDX 17	0.99482
PDX 47	0.99453

Supplemental Table 1. **Sample-level correlation between unfiltered and filtered RNA-Seq datasets**. List of r values for correlation comparisons of expression of the top 2,000 most variable genes for each PDX in the unfiltered RNA-Seq dataset (mouse and human mRNA reads) versus the filtered RNA-Seq dataset (only human reads).
