# Supplementary material for: Therapeutic implications of transcriptomics in head and neck cancer patient-derived xenografts
Source: PLoS One. 2023 Mar 1;18(3):e0282177. doi: 10.1371/journal.pone.0282177 (PMC9977000; doi:10.1371/journal.pone.0282177)
Supplement: S2 Table — List of r values for the 3.8% of transcripts (76/2000) with r <0.99 at the gene-level. Correlations are comparing expression level of each of the top 2,000 most variable genes across the PDXs before and after filtering for mouse reads. (PDF) [file pone.0282177.s002.pdf]

Ensembl ID	Gene Symbol	Gene-level correlation (r)
ENSG00000165194	*PCDH19*	0.98997
ENSG00000125378	*BMP4*	0.98989
ENSG00000133083	*DCLK1*	0.98956
ENSG00000198794	*SCAMP5*	0.98928
ENSG00000171766	*GATM*	0.98911
ENSG00000184588	*PDE4B*	0.98907
ENSG00000179148	*ALOXE3*	0.98882
ENSG00000104177	*MYEF2*	0.98876
ENSG00000182568	*SATB1*	0.98855
ENSG00000172020	*GAP43*	0.98839
ENSG00000162878	*PKDCC*	0.98837
ENSG00000066382	*MPPED2*	0.988
ENSG00000260027	*HOXB7*	0.98768
ENSG00000169122	*FAM110B*	0.98767
ENSG00000175567	*UCP2*	0.98764
ENSG00000104723	*TUSC3*	0.98743
ENSG00000206538	*VGLL3*	0.98738
ENSG00000101333	*PLCB4*	0.98713
ENSG00000198542	*ITGBL1*	0.9868
ENSG00000122592	*HOXA7*	0.98659
ENSG00000168916	*ZNF608*	0.98658
ENSG00000135097	*MSI1*	0.98637
ENSG00000154822	*PLCL2*	0.98634
ENSG00000156298	*TSPAN7*	0.98562
ENSG00000172915	*NBEA*	0.98551
ENSG00000187627	*RGPD1*	0.98536
ENSG00000132170	*PPARG*	0.98471
ENSG00000168542	*COL3A1*	0.98454
ENSG00000170837	*GPR27*	0.98438
ENSG00000259952	-	0.98431
ENSG00000197106	*SLC6A17*	0.984
ENSG00000155849	*ELMO1*	0.98361
ENSG00000170153	*RNF150*	0.9836
ENSG00000077063	*CTTNBP2*	0.98308
ENSG00000170689	*HOXB9*	0.98205
ENSG00000077782	*FGFR1*	0.9805
ENSG00000122756	*CNTFR*	0.97959
ENSG00000164692	*COL1A2*	0.97893
ENSG00000133134	*BEX2*	0.97841
ENSG00000135363	*LMO2*	0.97741
ENSG00000129682	*FGF13*	0.97732
ENSG00000183091	*NEB*	0.97718
ENSG00000085276	*MECOM*	0.97691
ENSG00000171004	*HS6ST2*	0.97645
ENSG00000198947	*DMD*	0.97614
ENSG00000182985	*CADM1*	0.97419
ENSG00000140092	*FBLN5*	0.97088
ENSG00000184304	*PRKD1*	0.97023
ENSG00000049192	*ADAMTS6*	0.97014
ENSG00000168386	*FILIP1L*	0.96919
ENSG00000124785	*NRN1*	0.96884
ENSG00000185811	*IKZF1*	0.96478
ENSG00000180818	*HOXC10*	0.96396
ENSG00000114405	*C3orf14*	0.96256
ENSG00000091656	*ZFHX4*	0.96242
ENSG00000147257	*GPC3*	0.9624
ENSG00000022267	*FHL1*	0.96237
ENSG00000104332	*SFRP1*	0.96037
ENSG00000124208	*TMEM189-UBE2V1*	0.95993
ENSG00000117707	*PROX1*	0.95817
ENSG00000006468	*ETV1*	0.95196
ENSG00000154864	*PIEZO2*	0.95164
ENSG00000108001	*EBF3*	0.95029
ENSG00000123338	*NCKAP1L*	0.94684
ENSG00000113140	*SPARC*	0.94271
ENSG00000151702	*FLI1*	0.94066
ENSG00000214575	*CPEB1*	0.93847
ENSG00000163017	*ACTG2*	0.93732
ENSG00000140937	*CDH11*	0.93177
ENSG00000196604	*POTEF*	0.90389
ENSG00000048740	*CELF2*	0.89724
ENSG00000233608	*TWIST2*	0.871
ENSG00000116132	*PRRX1*	0.82153
ENSG00000269955	*C7orf55-LUC7L2*	0.81838
ENSG00000254870	*ATP6V1G2-DDX39B*	0.80955
ENSG00000258017	-	0.47602

Supplemental Table 2. **Gene-level correlation between unfiltered and filtered RNA-Seq datasets**.
List of r values for the 3.8% of transcripts (76/2000) with r <0.99 at the gene-level. Correlations are comparing expression level of each of the top 2,000 most variable genes across the PDXs before and after filtering for mouse reads.
