# Supplementary material for: Therapeutic implications of transcriptomics in head and neck cancer patient-derived xenografts
Source: PLoS One. 2023 Mar 1;18(3):e0282177. doi: 10.1371/journal.pone.0282177 (PMC9977000; doi:10.1371/journal.pone.0282177)
Supplement: S3 Table — Gene Ontology (GO) annotated pathway analysis to assess for common biological functions between the 76 genes with gene-level correlation r values <0.99. Skeletal system development was the only GO term with q-value <0.5. (PDF) [file pone.0282177.s003.pdf]

categoryId	description	pValue	qValue	geneId	count
GO:0001501	skeletal system development	1.40E-05	0.019	*PKDCC*<br>*PRRX1*<br>*HOXA7*<br>*FGFR1*<br>*HOXC10*<br>*HOXB9*<br>*BMP4*<br>*HOXB7*<br>*CDH11*<br>*COL3A1*<br>*COL1A2*<br>*SFRP1*<br>*FLI1*	13

Supplemental Table 3. **Biological process pathway analysis of transcripts with lower gene-level correlation between unfiltered and filtered RNA-Seq datasets**.
Gene Ontology (GO) annotated pathway analysis to assess for common biological functions between the 76 genes with gene-level correlation r values <0.99. Skeletal system development was the only GO term with q-value <0.5.
