# Supplementary material for: Therapeutic implications of transcriptomics in head and neck cancer patient-derived xenografts
Source: PLoS One. 2023 Mar 1;18(3):e0282177. doi: 10.1371/journal.pone.0282177 (PMC9977000; doi:10.1371/journal.pone.0282177)
Supplement: S4 Table — Correlation of demographic and clinical characteristics of PDXs with cluster membership reveals that HPV status and tumor site are the only variables significantly correlated with hierarchical gene expression-based clustering (when partitioned by either 2, 3, or 4 clusters). Age, sex, and stage were not significantly associated with expression-based clustering in any analyses conducted. (PDF) [file pone.0282177.s004.pdf]

		P-value of association		
		2 clusters	3 clusters	4 clusters
**Variable**	Age	0.45	0.45	0.59
	Sex	1	0.82	0.8
	Stage (4 levels)	0.56	0.2	0.12
	Stage 3/4 vs. 1/2	0.56	0.16	0.21
	Stage 4 vs. 1/2/3	0.2	0.11	0.17
	HPV (sequencing)	*6.20E-08* *	*1.40E-07* *	*2.00E-15* *
	HPV (clinical)	*8.40E-07* *	*2.80E-06* *	*5.60E-12* *
	Site	*3.20E-08* *	*2.70E-07* *	*5.00E-09* *

Supplemental Table 4. **PDX cluster associations with patient demographic and tumor variables**. Correlation of demographic and clinical characteristics of PDXs with cluster membership reveals that HPV status and tumor site are the only variables significantly correlated with hierarchical gene expression-based clustering (when partitioned by either 2, 3, or 4 clusters). Age, sex, and stage were not significantly associated with expression-based clustering in any analyses conducted.
